# Comparison of Readmission, Discharge Location and Mortality over Three Years Post-Discharge Between Patients Diagnosed with Hospital-Acquired Malnutrition and Those Malnourished on Admission—A Retrospective Matched Case–Control Study in Five Facilities

**DOI:** 10.3390/healthcare13040364

**Published:** 2025-02-08

**Authors:** Breanne Hosking, Lynda Ross, Angela Vivanti, Sally Courtice, Amanda Henderson, Fiona Naumann, Rachel Stoney, Michelle Palmer

**Affiliations:** 1 Nutrition and Dietetics Department, Logan Hospital, Meadowbrook, QLD 4131, Australia; 2 Faculty of Health, Queensland University of Technology, Kelvin Grove, QLD 4059, Australia; 3 Nutrition and Dietetics Department, Princess Alexandra Hospital, Woolloongabba, QLD 4102, Australia; 4 School of Human Movement and Nutrition Studies, University of Queensland, St Lucia, QLD 4072, Australia; 5 Nutrition and Dietetics Department, Queen Elizabeth II Jubilee Hospital, Coopers Plains, QLD 4108, Australia; 6 Nursing Practice Development Unit, Princess Alexandra Hospital, Woolloongabba, QLD 4102, Australia; 7 Faculty of Nursing, Midwifery and Social Sciences, Central Queensland University, Brisbane, QLD 4000, Australia; 8 Nutrition and Dietetics Department, Beaudesert Hospital, Beaudesert, QLD 4285, Australia; 9 Nutrition and Dietetics Department, Redland Hospital, Cleveland, QLD 4163, Australia

**Keywords:** malnutrition, adult malnutrition, hospital-acquired malnutrition, discharge outcomes, discharge location, mortality, readmission

## Abstract

**Background/Objectives:** Increased mortality and poor post-discharge outcomes are common in malnourished inpatients. It is unknown whether post-discharge outcomes differ between patients with hospital-acquired malnutrition (HAM) or malnutrition present on admission (MPOA), which could impact nutrition processes within healthcare systems and hospital-acquired-complication policy. This retrospective matched case–control study compared mortality, discharge location and readmission at 3-, 12- and 36-months post-discharge between HAM and MPOA patients. **Methods:** The eligible patients were ≥18 years, malnourished and stayed in hospital for >14 days between 2015 and 2019. HAM patients were 1:1 matched with MPOA patients for age (±3 years), sex, facility and year of admission and further categorised by age group (18 < 65, ≥65 years). The data were obtained from medical records included demographics, mortality, discharge location and readmissions. Statistical tests were used to compare the groups. **Results:** There were 350 eligible patients (n = 175 HAM, 65 ± 18 years, 37%F, 88% moderately malnourished, 71% from hospitals with >500 beds). HAM and MPOA patients had similar post-discharge mortality (n = 51/175 (29%) vs. n = 64/175 (37%), *p* > 0.172) and discharge locations (n = 101/111 (81%) vs. n = 91/124 (82%) resided at home, *p* = 1.00) at 36 months. Of those readmitted to hospital (n= 268/350, 77%), days hospitalised post-discharge (HAM:17(6–40) vs. MPOA:19(8–39)) and number of readmissions (HAM:2(1–4) vs. MPOA:2(1–5)) were similar at 36 months (*p* > 0.05). However, older MPOA patients were more likely to readmit within 30 days (*p* = 0.007). **Conclusions:** Mortality was high but similar between MPOA and HAM patients up to 36 months post-discharge. Discharge location and readmissions were also similar between the groups, except that older MPOA patients were more likely to readmit to hospital within 30 days than older HAM patients. Mechanisms, such as nutrition policies and procedures, implementation of post-discharge nutrition interventions or allocation of post-discharge resources, should be explored further and should consider all long-stay malnourished patients, particularly those aged ≥ 65 years, to reduce preventable patient harm associated with malnutrition.

## 1. Introduction

The high prevalence and consequences of malnutrition in hospital inpatients are well established, including delayed recovery and wound healing, increased risk of pressure injuries, increased length of hospital stay and increased healthcare costs [1]. Malnourished patients may also have poorer outcomes post-discharge compared to well-nourished patients. This can include a higher risk of requiring care at home or admission to a care facility, readmitting to hospital within 30 days and higher mortality rates for up to three years post-discharge [1,2].

The vast published malnutrition literature has only recently made a distinction between hospital-acquired malnutrition (HAM) and malnutrition present on admission (MPOA). Malnutrition developing in adults during a hospital stay may have different contributing factors compared to that in community-dwelling adults, including acute inflammatory responses and disease state changes, which can reduce appetite and oral intake and increase muscle catabolism and weight loss [3]. Hospital inpatients also face individual and operational barriers that contribute to reduced nutritional intake and malnutrition development, such as mealtime interruptions, dissatisfaction with hospital food service, prolonged fasting periods, treatment effects and chewing difficulties [4].

Internationally, there is a lack of consensus on the definition of HAM [5]. A 2023 systematic review on HAM incidence included studies defining HAM as any nutritional status decline between two timepoints during admission independent of nutrition status on admission or any validated assessment method such as the Subjective Global Assessment or Mini Nutritional Assessment [5]. That review found a pooled HAM incidence of 25.9% in acute settings [5].

While nutrition and hydration are internationally acknowledged to play a role in moderating hospital-acquired complications (HACs) such as falls and pressure injuries, HAM is rarely listed as an HAC [6,7]. Within Australia, HAM has been recognised by the Australian Commission in Quality and Safety in Health Care (ACQSHC) as an HAC since 2018 [8]. The ACQSHC defines malnutrition according to the International Classification of Disease Australian Modification (ICD-AM) [9]. Hospital-acquired malnutrition, in this study, is defined as malnutrition that first develops more than 14 days after hospital admission, aligning with the ACQSHC HAC and ICD-AM definitions. Using this more precise definition, recent prospective studies have found a HAM prevalence between 4.5 and 28.3% [10,11].

Currently, there is a paucity of post-discharge outcome data internationally for patients with HAM, including mortality rates, discharge location and readmission rates. A 2020 Brazilian study found that patients with a deterioration in nutrition status in the first week of hospitalisation were more likely to readmit 6 months post-discharge [12]. No other studies were located comparing post-discharge outcomes between patients with HAM and those with MPOA. The longer-term trajectory of patients with HAM is unknown, including discharge location and mortality and whether these differ from those for MPOA patients.

This study, therefore, aimed to compare the rates of readmission, discharge location and mortality at 3, 12 and 36 months post-discharge between patients with HAM and those diagnosed with MPOA. The results may improve the understanding of HAM and its longer-term impact on patient outcomes and healthcare use to assist with nutrition care policy planning including the allocation of nutrition resources.

## 2. Materials and Methods

### 2.1. Study Design, Setting and Ethics

This study was a retrospective case–control matched study of malnourished patients admitted to one of five facilities within Southeast Queensland, Australia. One hospital was a 1000-bed tertiary metropolitan hospital, two were medium secondary metropolitan hospitals with 270–450 beds and two were smaller regional hospitals with 30–180 beds. The retrospective case–control matched design offered an efficient and feasible way to capture a three-year follow-up period and an opportunity to assess differences if numbers were limited.

This study is exploratory in nature, and it was not possible to perform a power calculation given there is only one study [12] internationally that compares post-discharge outcomes (mortality and discharge location at 6 months only) between HAM and MPOA patients.

Ethical clearance was obtained prior to study commencement (HREC/2021/QMS/77453). A waiver of consent was approved given the retrospective study design. The definition of HAM used in this study was malnutrition first diagnosed more than 14 days after hospital admission [13,14].

### 2.2. Patient Eligibility

The HAM patient cohort was identified in a previous study [13]. In this study, these HAM cases were 1:1 matched with controls from the same malnourished patient cohort who had malnutrition present on admission (MPOA). Case–control matching was based on age (±3 years), sex, facility and year of admission. Each case and control were selected once only. This same malnourished patient cohort that both HAM and MPOA patients were originally selected from comprised adults aged ≥ 18 years, admitted to and discharged from one of the five facilities described above between 1 July 2015 and 31 January 2019, who had a length of stay (LOS) > 14 days and were documented as malnourished during the admission by either the dietitian or the medical officer and were coded as malnourished by clinical coders using standardised criteria from the *International Statistical Classification of Diseases*, *Tenth Revision*, *Australian Modification* (ICD-10-AM) [13]. Patients with HAM were identified as those whom clinical coders had coded with “malnutrition that arose during admission” and who were clinically judged by dietitians to have had malnutrition that was first diagnosed more than 14 days after admission and was not present on admission [13]. Eligible patients were admitted to any ward, including the intensive care, acute, sub-acute or mental health wards [13]. Patients were excluded if they did not meet the eligibility criteria previously described [13] and were additionally excluded if they passed away or received palliative care during the index admission.

### 2.3. Data Collection

The demographic, clinical, hospital admission, mortality, discharge location and hospital readmission and re-presentation data were sourced from statewide public hospital electronic medical records. The demographic data included age at admission and sex. The clinical data included malnutrition severity (moderate (B)/severe (C)) according to the Subjective Global Assessment [15] as determined by a dietitian. The mortality data included date of death (if recorded) to determine whether patients had subsequently passed away within 3, 12 or 36 months of the index admission. Patients were assumed to be alive if no date of death was recorded. The hospital admission data included facility size (categorised as <200, 200–500 or >500 beds) and LOS of the index admission. The discharge location data included discharge location (community dwelling or residential care facility) initially post-discharge and at 3, 12 and 36 months post-discharge. The hospital re-presentation and readmission data included the number of public hospital emergency department re-presentations and public hospital readmissions, including planned or emergency admissions, and the dates of re-presentation, readmission and discharge (to calculate time to re-presentation or readmission and LOS).

### 2.4. Data Analysis

All the data were entered into Microsoft Excel version 16.13.1 (Microsoft Corp., Redmond, WA, USA) and analysed in SPSS version 29.0 (IBM Corp., Armonk, NY, USA). Case–control matching was performed using the case–control matching function in SPSS. Descriptive analyses were reported as proportions of the total (n, %) and mean ± SD or the median and interquartile range. The two groups compared were HAM and MPOA, and they were further analysed according to age category (either aged 18–65 or ≥65 years). Continuous data were tested for normality, with independent sample t-tests used to compare parametric data between groups and Mann–Whitney U-tests for non-parametric data. Pearson’s chi-squared and Fisher’s exact tests were used to compare categorical data between groups. Statistical difference was classified as *p* < 0.05. Variables approaching significance differences (*p* < 0.2) between those readmitted and not readmitted within 30 days were included in a binary logistic regression analysis using a backwards Wald selection method to determine factors associated with being readmitted within 30 days of the index admission.

## 3. Results

While 208 patients were previously identified with HAM [13], 33 were excluded given they had passed away (n = 26) or were admitted for palliative care (n = 7) during the index admission, leaving 175 HAM patients to 1:1 match with MPOA controls. Matching was successful by age (±3 years), sex, facility and year of admission (Table 1). Most matched pairs had the same severity of malnutrition (77%, n = 135/175 pairs).

Most patients were male (63%), admitted to the largest facility (71%), moderately malnourished (88%) and 65 ± 18 years of age (Table 1). No demographic, clinical or hospital admission data differences were observed between the HAM and MPOA groups, except that patients with HAM had a longer LOS during the index admission (*p* < 0.001), as noted previously [13].

By 36 months post-discharge, one-third (33%) of the total cohort had passed away, 18% were living in a residential care facility, 54% had re-presented to the emergency department of a public hospital and 77% had been readmitted to a public hospital. Patients who re-presented to emergency departments had a median of two re-presentations. Patients readmitting to hospital had a median of two readmissions and stayed a median of 18 days.

Mortality, discharge location and readmissions were similar between the HAM and MPOA groups over 36 months post-discharge (*p* > 0.05), except that more HAM patients had planned readmissions (33%) than MPOA patients (22%) (*p* = 0.031). While the median days to first hospital readmission were statistically similar between groups (*p* = 0.254), the median number of days was 68 in MPOA patients and 99 in HAM patients, which may be clinically significant. Mortality, discharge location and readmissions were generally similar between the HAM and MPOA groups when further analysed by age category (18–65 or ≥65 years) (Supplementary Table S1). However, older MPOA patients also showed a trend of being more likely to have passed away by 36 months than older HAM patients (*p* = 0.063). Older (≥65 years) MPOA patients were more likely to readmit within 30 days of discharge (*p* = 0.007) and showed a trend of being readmitted 61 days sooner than older HAM patients by (*p* = 0.079).

Regression analysis showed that patients who passed away by 3 months after the index admission were four times more likely to readmit within 30 days (*p* < 0.001, Table 2). There was also a trend that older patients (OR 0.618, *p* = 0.063) and patients with HAM (OR 0.637, *p* = 0.081) were less likely to readmit within 30 days of discharge.

## 4. Discussion

This is the first known study internationally to compare the post-discharge outcomes of patients with HAM to those of patients with MPOA over three years. Mortality, discharge location and readmissions were similar between patients with HAM and with MPOA matched by age (±3 years), sex, facility and year of admission at 3, 12 and 36 months post-discharge. There was also a trend that older patients (OR 0.618, *p* = 0.063) and patients with HAM (OR 0.637, *p* = 0.081) were less likely to readmit within 30 days of discharge.

### 4.1. Mortality

Patients with HAM from the same cohort as this study were previously shown to have a higher mortality than MPOA patients during their index admission [13]. The mortality rates of all patients with HAM and MPOA were similar up to three years post-discharge. The post-discharge mortality in this study could be understated if the patient’s medical record was not up to date. This is an acknowledged limitation of the retrospective study design, which relies on data available in the medical records. However, the risk is likely low given the three-year timeframe and that electronic medical records are shared across many public healthcare facilities in the state.

The only comparable study located also found that mortality at six months post-discharge was similar in Brazilian long-stay patients (>7 days) who experienced nutritional decline (20%, n = 3) or experienced no decline (15%, n = 17) [12]. The similar mortality between groups may be due to similar factors contributing to the development of malnutrition and/or similar barriers to optimal nutrition care.

There was a trend showing more older MPOA patients passing away 36 months after discharge. Advancing age is a recognised, non-modifiable factor increasing the risk of malnutrition [10]; however, this trend suggests there may be differences between older HAM and MPOA patients impacting mortality rate. This may include other risk factors contributing to malnutrition development (such as disease, food access or social support) and/or malnutrition management [4]. It was beyond the scope of this study to investigate the aetiology of malnutrition in the patient groups; however, the quality of nutrition care received by this HAM cohort has been previously investigated [14].

A 2022 systematic review highlighted that post-discharge nutrition support reduced mortality in community-dwelling malnourished adults [16]. Interventions included oral nutrition supplementation and/or dietary advice including specific individualised energy and protein intake recommendations [16]. Implementation of post-discharge models of care using these effective malnutrition management strategies may help reduce mortality.

### 4.2. Discharge Location

The proportion of patients either living at home or at a residential care facility post-discharge was similar between HAM patients and those malnourished on admission at each time point over the three years post-discharge. While there is no previous literature distinguishing the rates of HAM and MPOA patients residing in a residential care facility, malnourished patients are more likely to discharge to a higher level of care than well-nourished patients [17]. Approximately one in five malnourished patients in our study were living in a residential care facility by 36 months. Other malnutrition studies have shown similar rates of 18–50% [18,19]. Targeted nutrition interventions for malnourished community-dwelling and residential aged care facility residing older adults may improve nutritional intake and body weight [16,20].

### 4.3. Readmission

Hospital readmission data were generally similar across three years post-discharge between the HAM and MPOA groups. The only similar study located found that Brazilian patients with a deterioration in nutrition status in the first week of hospitalisation were more likely to readmit within 6 months post-discharge compared to well-nourished or stable chronically malnourished patients [17,20]. The differences in these findings may be explained by the different definitions of HAM and the timeframes for HAM to develop (i.e., one week vs. two weeks), on which there is currently no global consensus [5]. There could also be differences in the nutrition care provided during admission and post-discharge, which may impact readmission.

More HAM patients had planned readmissions (33%) than MPOA patients (22%) (*p* = 0.031); however, the median number of emergency department presentations, readmissions and number of days hospitalised were similar between the groups up to three years post-discharge. This finding suggests that the overall hospital service utilisation of HAM and MPOA patients may not be clinically significant in the longer term post-discharge.

The regression analysis also showed a trend for patients with MPOA to be more likely to readmit within 30 days, independent of age and those passing away within three months. More older (aged ≥65 years) MPOA patients were readmitted to hospital within 30 days of discharge than older HAM patients. Older malnourished patients, in general, may have increased hospital readmission rates than well-nourished patients. The hospital emergency presentation and readmission data could be understated as patients may have accessed private hospital or interstate public hospital services not captured by this study. However, this limitation is mitigated given public hospitals have a greater proportion (59%) of total hospital admissions in Australia [21]; and, generally, publicly and privately funded patients in Australia consistently access healthcare within the public and private sectors, respectively [22,23].

Chronically malnourished patients may be more vulnerable and have a risk of acute on chronic deterioration, which may impact their healthcare utilisation in the short term including hospital readmission. Supportive care may be beneficial to all adult HAM and MPOA patients post-discharge to reduce chances of hospital readmission, with opportunities to further target older MPOA patients. Models of care that target the transition of older malnourished adults from hospital to home can improve nutritional status [24,25].

While it appears that patients with HAM and MPOA have generally similar post-discharge outcomes, HAM is a hospital-acquired complication that occurs while patients are under the direct nutrition care of a hospital service. Previous studies found that 70% of HAM cases may be preventable [26] and that malnutrition screening and management remains less than ideal in HAM patients [14]. Therefore, the health service has a duty of care to ensure that nutrition screening, assessment and management systems are optimised to reduce preventable harm to patients, with an emphasis placed on long-stay patients.

### 4.4. Strengths, Limitations and Future Directions

A strength of this study was that the case–control matching was successful. While this study did not match for malnutrition severity, few (12%) patients had severe malnutrition, and only 27% of patients were not matched by malnutrition severity. The limitations of hospital clinical data also meant that some demographic data, such as ethnicity or income, were unable to be collected. Future studies could consider broader demographic data as well as matching by malnutrition severity. The three-year post-discharge follow-up period was another study strength as the only other previously comparable study had a follow-up period of six months [12].

The definition of HAM used in this study (i.e., decline from well nourished on admission to moderately or severely malnourished) is clearly defined and aligns with malnutrition and HAC definitions. An alternative definition of HAM as any nutritional decline during hospital admission regardless of nutrition status on admission is also used in the literature. Consideration should be given to the HAM definition used when comparing this study’s results to others; however, the impact may be small, with a recent prospective study finding the prevalence of HAM and nutritional decline during a longer-stay admission (>7 day) to be similar (28.3% and 25.4%, respectively) [11].

While this study was completed in adult inpatients across multiple wards within five facilities ranging in size and location, all these facilities were located within the same health service district with shared or similar policies and procedures on inpatient nutrition management. Ford et al. (2024) [27] suggest that “policy can be a lever to accelerate nutrition care best practices”. Strong policy recognition of HAM at the district (local policies and procedures) and national (HAM recognised as a HAC) levels could explain the lack of differences in post-discharge outcomes if HAM cases are generally identified and receive nutrition care. Care should be taken when generalising these results to other health services in which inpatient nutrition screening, assessment and management policies and systems may not be in place or well applied.

No power calculation was able to be conducted due to the lack of existing literature. Insufficient power may be a reason that no differences were observed between groups for most variables. However, the sample size was large (n = 350), and the data are now available for future studies to conduct appropriately powered studies.

It was beyond the scope of this study to investigate the aetiology of malnutrition in each patient group, which could include presenting condition, disease history and socioeconomic factors [3]. Further studies could investigate whether there are differences in post-discharge outcomes between acute or chronic disease-related malnutrition (with or without inflammation) and starvation-related malnutrition rather than between HAM or MPOA [3]. Further work is also required regarding effective nutrition interventions for the prevention and management of HAM.

## 5. Conclusions

This retrospective matched case–control study found that the mortality, discharge location and readmission of patients with hospital-acquired malnutrition were high but similar to those of patients who were malnourished on admission at 3, 12 and 36 months post-discharge. Readmissions were also similar between the groups, except that older MPOA patients were more likely to readmit to hospital within 30 days than older HAM patients. Mechanisms, such as nutrition policies and procedures, implementation of post-discharge nutrition interventions or allocation of post-discharge resources, should be explored further and should consider all long-stay malnourished patients, particularly those aged ≥ 65 years, to reduce preventable patient harm associated with malnutrition.

## Figures and Tables

**Table 1:** Demographic, clinical, hospital admission, mortality, discharge location and readmission descriptors compared between those with hospital-acquired malnutrition (HAM) and malnutrition present on admission (MPOA).

Outcome Measure	Total n = 350 n (%)	MPOA (Control) n = 175 n (%)	HAM (Case) n = 175 n (%)	*p* Value
**Demographic and clinical data**				
Age at admission, mean± SD	65 ± 18	65 ± 18	65 ± 18	0.699 ^t^
Sex, female	128 (37)	64 (37)	64 (37)	1.000
Malnutrition severity • Moderate • Severe	308 (88) 42 (12)	151 (86) 24 (14)	157 (90) 18 (10)	0.411
**Hospital admission data**				
Facility size • >500 beds • 200–500 beds • <200 beds	248 (71) 86 (25) 16 (5)	124 (71) 43 (25) 8 (5)	124 (71) 43 (25) 8 (5)	1.000
Length of stay (index admission), days, median (IQR)	22 (16–54)	16 (15–17)	51 (31–85)	**<0.001** ^mw^
Mortality post-discharge *				
• 3 months • 12 months • 36 months	26 (7) 63 (18) 115 (33)	16 (9) 36 (21) 64 (37)	10 (6) 27 (15) 51 (29)	0.308 0.266 0.172
**Discharge location**				
Post-initial discharge • Home • Residential care facility • Other hospital	278 (79) 46 (13) 26 (7)	143 (82) 19 (11) 13 (7)	135 (77) 27 (15) 13 (7)	0.471
3 months post-discharge (n = 324:165/159) ^#^ • Home • Residential care facility	270 (83) 54 (17)	138 (87) 21 (13)	132 (80) 33 (20)	0.135
12 months post-discharge (n = 287:148/139) ^#^ • Home • Residential care facility	239 (83) 48 (17)	118 (85) 21 (15)	121 (82) 27 (18)	0.528
36 months post-discharge (n = 235:124/111) ^#^ • Home • Residential care facility	192 (82) 43 (18)	91 (82) 20 (18)	101 (81) 23 (19)	1.000
**Hospital readmission and re-presentation across 36 months**				
Number who re-presented to public hospital EDs post-discharge	189 (54)	97 (55)	92 (53)	0.668
Of those who re-present to public hospital EDs post-discharge, number of times re-presenting per patient, median (IQR) (n = 189)	2 (1–3)	2 (1–3)	2 (1–4)	0.105 ^mw^
Number who readmitted to a public hospital post-discharge	268 (77)	134 (77)	134 (77)	1.000
Number of patients who readmitted to a public hospital for emergency readmissions	238 (68)	121 (69)	117 (67)	0.731
Number of patients who readmitted to a public hospital for planned readmissions	97 (28)	39 (22)	58 (33)	**0.031**
Of those who readmit, number public hospital readmissions per patient, median (IQR) (n = 268:134/134)	2 (1–5)	2 (1–5)	2 (1–4)	0.448 ^mw^
Of those who readmit, number of emergency public hospital readmissions per patient, median (IQR) (n = 238:117/121)	2 (1–4)	2 (1–5)	2 (1–4)	0.864 ^mw^
Of those who readmit, number of planned public hospital readmissions per patient, median (IQR) (n = 97:58/39)	1 (1–2)	1 (1–2)	1 (1–2)	0.662 ^mw^
Of those who readmit, total number of days hospitalised in public hospital for readmissions per patient, median (IQR) (n = 268:134/134)	18 (6–40)	19 (8–39)	17 (6–40)	0.661 ^mw^
Of those who readmit, days to first public hospital readmission per patient, median (IQR) (n = 268:134/134)	82 (20–293)	68 (15–315)	99 (29–272)	0.254 ^mw^
Of those who readmit, number readmitting to a public hospital within 30 days of discharge (n = 268:134/134)	85 (32)	50 (37)	35 (26)	0.066
Of those who readmit, number readmitting to a public hospital within 90 days of discharge (n = 268:134/134)	140 (52)	76 (57)	64 (48)	0.178

**Bold** *p* values identify statistical significance (*p* < 0.05). When sample sizes have changed due to a subset of data being selected, they are reported as (total:MPOA/HAM). Statistical tests are Pearson’s chi-squared tests unless otherwise specified. * Totals are cumulative from previous timepoints. ^#^ Sample sizes reduced due to patients’ passing away. ED, emergency department; IQR, interquartile range (Q1–Q3); ^mw^, Mann–Whitney U-test; ^t^, *t*-test.

**Table 2:** Binary logistic regression backwards Wald analysis of factors associated with adult inpatients who were readmitted within 30 days (versus those who were not readmitted within 30 days).

Variable	Odds Ratio	95% CI for OR		*p* Value
		Lower	Upper	
HAM (vs. MPOA)	0.637	0.383	1.057	0.081
Passed away by 3 months	4.285	1.868	9.828	**<0.001**
Age ≥65 years (vs. 18–65 years)	0.618	0.372	1.027	0.063
Constant	0.589	-	-	0.013

**Bold** *p* values identify statistical significance (*p* < 0.05). Sample size included in analysis n = 350. Nagelkerke R square 0.073, sensitivity 63%, specificity 78%. Other variables included in the regression analysis but not significantly associated with readmission within 30 days: length of stay of index admission. CI, confidence interval; HAM, hospital-acquired malnutrition; MPOA, malnutrition present on admission; OR, odds ratio. Due to the contradiction between discharge location and passing away at 3 months, it was necessary to run two models, and the better performing model that excluded discharge location from the analysis is reported here based on Nagelkerke R squared, overall agreement and sensitivity and specificity.

## Data Availability

The data in this study are available on request from the corresponding author.

## Supplementary Materials

The following supporting information can be downloaded at: https://www.mdpi.com/article/10.3390/healthcare13040364/s1, Table S1: Mortality, discharge location and readmission descriptors compared between those with hospital-acquired malnutrition (HAM) and malnutrition present on admission (MPOA) categorised according to age (18–65 years versus ≥65 years).
