# Supplementary material for: Comparison of Readmission, Discharge Location and Mortality over Three Years Post-Discharge Between Patients Diagnosed with Hospital-Acquired Malnutrition and Those Malnourished on Admission—A Retrospective Matched Case–Control Study in Five Facilities
Source: Healthcare (Basel). 2025 Feb 8;13(4):364. doi: 10.3390/healthcare13040364 (PMC11855929; doi:10.3390/healthcare13040364)
Supplement: Supplementary file 1 [file healthcare-13-00364-s001.zip › healthcare-3316767-supplementary.pdf]

Table S1: Mortality, discharge location and readmission descriptors compared between those with hospital acquired malnutrition (HAM) and malnutrition present on admission (MPOA) categorised according to age (18-<65 years versus ≥65 years)

Outcome measure	Age 18-<65 years				Age ≥65 years			
	Total (n=144/350) n (%)	MPOA (control) (n=68) n (%)	HAM (case) (n=76) n (%)	*p* value	Total (n=206/350) n (%)	MPOA (control) (n=107) n (%)	HAM (case) (n=99) n (%)	*p* value
**Mortality post discharge***								
3 months	8 (6)	5 (7)	3 (4)	0.476 [f]	18 (9)	11 (10)	7 (7)	0.467
12 months	20 (14)	10 (15)	10 (13)	0.814	43 (21)	26 (24)	17 (17)	0.233
36 months	34 (24)	15 (22)	19 (25)	0.699	81 (39)	49 (46)	32 (32)	0.063
**Discharge location**								
Discharge location - 3 months post-discharge # (<65 n=136:63/73, ≥65 n=188:96/92)								
Home	132 (97)	62 (98)	70 (96)	0.624[f]	138 (73)	76 (79)	62 (67)	0.072
Residential Care Facility	4 (3)	1 (2)	3 (4)		50 (27)	20 (21)	30 (33)	
Discharge location - 12 months post-discharge # (<65 n=124:58/66, ≥65 n=163:81/82)								
Home	120 (97)	57 (98)	63 (96)	0.622[f]	119 (73)	61 (75)	58 (71)	0.597
Residential Care Facility	4 (3)	1 (2)	3 (5)		44 (27)	20 (25)	24 (29)	
Discharge location - 36 months post-discharge # (<65 n=110:53/57, ≥65 n=125:58/67)								
Home	106 (96)	52 (98)	54 (95)	0.619[f]	86 (69)	39 (67)	47 (70)	0.847
Residential Care Facility	4 (4)	1 (2)	3 (5)		39 (31)	19 (33)	20 (30)	
**Hospital readmission and re-presentation across 36 months**								
Number who re-presented to public hospital EDs post-discharge	84 (58)	39 (57)	45 (59)	0.866	105 (51)	58 (54)	47 (48)	0.403
Of those that represent, number public hospital ED presentations per patient, median (IQR) (<65 n=84:39/45, ≥65 n=105:58/47)	2 (1-4)	2 (1-3)	2 (1-5)	0.455[mw]	2 (1-3)	1 (1-3)	2 (1-4)	0.173[mw]
Number who readmit to a public hospital post-discharge	114 (79)	53 (78)	61 (80)	0.838	154 (75)	81 (76)	73 (74)	0.751
Number patients with emergent readmissions to a public hospital post-discharge	97 (67)	45 (66)	52 (68)	0.782[mw]	141 (68)	76 (71)	65 (65)	0.430[mw]
Number patients with planned readmissions to a public hospital post-discharge	53 (37)	20 (55)	33 (43)	0.700[mw]	44 (21)	19 (18)	25 (25)	0.346[mw]

Of those that readmit, number total public hospital readmissions per patient, median (IQR) (<65 n=114:53/61, ≥65 n=154:81/73)	2 (1-5)	2 (1-5)	3 (2-5)	0.288^{mw}	2 (1-4)	2 (1-4)	2 (1-3)	0.910^{mw}
Of those that readmit, number emergency public hospital readmissions per patient, median (IQR) (<65 n=97:45/52, ≥65 n=141:76/65)	2 (2-5)	2 (1-6)	3 (2-5)	0.782^{mw}	2 (1-3)	2 (1-4)	1 (1-3)	0.430^{mw}
Of those that readmit, number planned public hospital readmissions per patient, median (IQR) (<65 n=53:20/33, ≥65 n=44:19/25)	1 (1-2)	1 (1-2)	1 (1-2)	0.700^{mw}	1 (1-2)	1 (1-3)	1(1-2)	0.346^{mw}
Of those that readmit, days hospitalised in public hospital across 36 months per patient, median (IQR) (<65 n=114:53/61, ≥65 n=154:81/73)	23 (8-55)	23 (6-54)	24 (9-58)	0.774^{mw}	16 (6-32)	17 (8-35)	12 (6-32)	0.254^{mw}
Of those that readmit, days to first public hospital readmission per patient, median (IQR) (<65 n=114:53/61, ≥65 n=154:81/73)	71 (17-300)	69 (14-331)	81 (18-232)	0.894^{mw}	92 (27-291)	66 (15-257)	127 (37-340)	0.079^{mw}
Of those that readmit, number of patients readmitting to a public hospital within 30 days of discharge (<65 n=114:53/61, ≥65 n=154:81/73)	41 (36)	19 (36)	22 (36)	1.000	44 (29)	31 (38)	13 (18)	**0.007**
Of those that readmit, number of patients readmitting to a public hospital within 90 days of discharge (<65 n=114:53/61, ≥65 n=154:81/73)	64 (56)	31 (59)	33 (54)	0.707	76 (49)	45 (56)	31 (43)	0.110

Bold *p* values identify statistical significance ($p<0.05$). When sample sizes have changed due to subset of data being selected, they are reported as (n=total:MPOA/HAM). Statistical tests are Pearson's chi squared tests unless otherwise specified. * totals are cumulative from previous timepoints. # sample sizes reduced due to patients passing away. ED emergency department, ^{f} fisher's exact test, IQR interquartile range (Q1-Q3), ^{mw} Mann-Whitney U-test, ^{t} t-test.
